# Challenges of establishing a Community Advisory Board (CAB) in a low-income, low-resource setting: experiences from Bagamoyo, Tanzania

**DOI:** 10.1186/1478-4505-7-16

**Published:** 2009-06-17

**Authors:** Kafuruki Shubis, Omar Juma, Rahiya Sharifu, Brandt Burgess, Salim Abdulla

**Affiliations:** 1 Ifakara Health Institute-Bagamoyo Research and Training Centre, Bagamoyo, United Republic of Tanzania

## Abstract

**Objective:**

Community Advisory Boards are now seen as standard practice for clinical vaccine and drug trials worldwide. In the past, most Community Advisory Boards (CABs) were established by activists and lobbyists to monitor HIV/AIDS vaccine and drug trials in developed countries. In Africa the first CAB was established in Uganda in 1990 in conjunction with an HIV vaccine project and has since been followed by others in South Africa, Zimbabwe, and Kenya. In 2007, the Bagamoyo branch of the Ifakara Health Institute initiated the formation of a CAB. The aim was to properly educate and empower elected CAB members to become full partners in all research activities concerning the public within the Bagamoyo area.

**Methods and Results:**

Beginning in 2007, staff visited each of the 24 villages within the study area to inform the communities about the proposed CAB and asked them to elect two individuals to represent their village on the CAB. The first attempt was hampered by community leaders selecting themselves, which led to inconsistent attendance, gender imbalance, and political infighting. New criteria for the selection of representatives were implemented to exclude governmental leaders, illiterate representatives and to promote a one-to-one gender balance. The newly appointed representatives underwent training and have participated in CAB meetings largely devoid of the negative issues previously encountered.

**Conclusion:**

The successfully established CAB has led to improved relations with the community and facilitated the recruitment of study subjects. Our experiences show that, it is possible to establish a non-specific CAB in a low-income setting.

## Background

In the late 1980's, concerned citizens and activists set about creating coalitions of community members seeking a voice in the HIV/AIDS research activities being conducted within their communities [1-6]. These groups, the early incarnations of today's Community Advisory Boards (CAB), succeeded in expressing their opinions and eventually changed the drug approval process within the United States [1-4]. CABs are now accepted as integral parts of most HIV/AIDS clinical trails worldwide [7-10]. The formation of CABs is seen as the best approach for research institutions to engage the community on issues pertaining to the research activities in the public realm [11-13]. This partnership is viewed as universally beneficial as the research institution receives much needed community support and the community ensures it is informed and its concerns are addressed by the researchers [11-13].

The Bagamoyo Branch (BRTC) of the Ifakara Health Institute (IHI) was established in 2005 as a site for malaria drug and vaccine clinical trials and is currently expanding into tuberculosis and HIV/AIDS related research. As much of our research requires the participation of many members of the public, we identified a need to facilitate community involvement and cooperation. We recognized that establishing a CAB could serve many important purposes, such as: ensuring a venue for expression of the community's concerns and feedback, aiding in the dissemination of results and information, enhancing consenting and follow up processes during clinical trials, helping manage false rumours, and ensuring proper crisis management.

This paper describes the processes and challenges encountered during the establishment of a continuous, non-specific CAB in the rural setting of Bagamoyo, Tanzania.

## Community demographics

The BRTC is located on the grounds of the Bagamoyo District Hospital, on the coast of the Indian Ocean about 70 kms north of Dar-es-Salaam, Tanzania. The BRTC employs almost 100 staff, including administrative support staff, clinicians and scientists. The BRTC study area covers 1160 km^2 ^and contains 89,000 people (2002 Tanzanian census) living in 24 villages. The village farthest from the IHI-Bagamoyo head office is about 58 km away. It takes the members from that village approximately 45 minutes by public transport (bus/mini-bus) to reach the meeting site.

Diverse ethnic groups including the Zaramo, Kwere, Doe and Zigua inhabit the study area and the majority are either subsistence farmers cultivating rice, maize and cassava or fisherman working on the Indian Ocean or the Ruvu River and its tributaries. Agriculture employs 76% of the population [14]. The adult literacy rate is greater than 70% [14]. Swahili, the national language, is spoken widely throughout the study area [14].

## Local governmental and administrative structure

In Tanzania, the topmost administrative level is the District Council (DC). The next two levels of administration beneath the DC consist of the Division and the Ward. Lastly, at the grass roots level are the village governments, formed by representatives elected from the hamlets within each village. The Bagamoyo study area has a total of 3 Divisions, 8 Wards, and 24 village governments.

## Establishment of the Bagamoyo District Community Advisory Board

The process for establishing a Community Advisory Board began when BRTC first held a series of meetings with members of the Bagamoyo District Council. A small team of dedicated BRTC researchers and clinicians acted as the primary representatives of the Centre throughout this and all subsequent community interactions. These meetings and discussions were in line with a Memorandum of Understanding between BRTC and Bagamoyo District Council established during the inception of BRTC that allowed us to enter into partnership with the villages of Bagamoyo District.

Due to the fact that the people were initially unaware of any opportunity to contribute to the research activities in their community and often believed the researchers were merely using the community as a resource, we viewed community sensitization on the importance and value of the CAB as the first and most critical step. Advocacy and sensitization meetings were conducted in every hamlet of the 24 villages through cooperation with village governments. In the first step, we met with village/hamlet leaders who then convened general village meetings. The BRTC field section was instrumental in this activity, as we were able to sensitize more than 4 villages a day by convening and conducting them at one location, as some villages are located close together geographically.

At the village meetings we introduced the concept of the CAB, its role, its importance in relation to the research at BRTC and the effects it would have in their communities. During these meetings, the community raised issues they wanted addressed by the researchers: payment for participating in studies, improvement of the quality of care, and care of study volunteers. These concerns were evidence that the community needed a formal and direct link to the BRTC. The BRTC staff then asked the communities to select two members to represent them on the CAB. Initially, twenty-four members from the various villages were selected to serve on the first CAB.

The newly elected representatives were almost exclusively male members from the various village governments. This group proved difficult to work with in our setting. Attendance was erratic with attendance rates of below 40%. This was a large problem as we viewed attendance as an absolute requirement for proper training and to ensure that the members were properly representing their villages during meetings. We often saw elected members send stand-in representatives to the meeting in their place. In addition to the problem of disrupting proper training, this defeated our attempt of establishing each member as an easily recognizable individual within the community as the person to whom members of the villages should approach with concerns and/or inquiries.

In addition to the attendance issues, we encountered problems that arose from differences in the political party affiliations of several of the members. At times, it was obvious politics were hindering cooperation and overriding the best interests of the community at large. Lastly, the gender imbalance (19 male, 5 female) of the CAB was viewed as unacceptable. BRTC's research involves mostly women and children as trial participants, so we believed that it was essential to seek strong female participation and perspective in all discussions.

After encountering these difficulties, we quickly came to the conclusion that the current make-up of the CAB had little chance of properly serving its functions as a bridge between BRTC and the community. We believed that the current CAB had to be disbanded and reformed. We realized that we needed to alter the make-up of the members elected to serve on the CAB. To achieve this, we new defined a new set of selection criteria. The new membership requirements prohibited selection of governmental leaders, sought gender balance (1:1, male to female), and required literacy (primary education). A willingness to do voluntary work and an interest in health matters were also stressed. We also decided to increase the number of elected representatives from each village from one to two to ensure a gender-balanced board. We then re-approached the communities and asked them to elect two new representatives according to these new criteria. Due to the fact that IHI-BRTC representatives were not present during the election of the new CAB members, we cannot detail how the various villages selected their representatives. We only know that the village leaders were responsible for imposing the new selection criteria on the election process.

After 2 weeks, all the villages had submitted the names of their newly elected representatives. Based on the meetings since the re-election, it appeared that the application of the new selection criteria to the selection of CAB members was successful. Meeting attendance improved to better than 98%, continuity of training was observed, the notion of ruling versus opposition party politics were eliminated in the group activities and community dealings, and gender balanced was achieved (24 male and 24 female). Since then, the Bagamoyo District CAB has been operating well.

## Creation of CAB training material

The CAB training material was prepared by BRTC scientists and IHI-Internal Review Board (IRB) secretariat members. The materials were initially created in English, then translated into Swahili, the universally spoken language in the study area. Translated versions were subjected to a verification process by BRTC scientists to ensure they retained the proper meaning and context of the original English version. Translation of the original English material into Swahili was, at times, difficult; it generated interesting discussions amongst the researchers on how to best convey scientific terms and concepts in Swahili. In these instances, following much discussion, a satisfactory translation was agreed upon by the researchers and IRB members.

## Training

The new CAB was inaugurated on August 17, 2007. During the first year of the reformed CAB, we conducted six training sessions to initiate the new members to the novel ideas of a Community Advisory Board and community involvement in scientific research. During the initial meeting, we facilitated election of the board chairperson and secretary and helped formulate the basic guidelines governing the conduct of CAB matters, i.e. frequency of meetings, how to communicate among the members and with BRTC and researchers, and financing of CAB activities. The next meeting was an orientation on IHI-BRTC (history, organizational structure and activities) and the role of health research in development. The third meeting covered the roles of communities in research and the role to be played by the CAB. The fourth session covered fundamental principles of research ethics, such as the concept of autonomy as applied through Informed Consent Forms (IFCs). The fifth training session was on methods of ensuring proper communication and overcoming inevitable barriers. Additionally, the CAB met with the IHI internal review board to discuss items pertaining to ICF content and better the interaction between the CAB representatives and the research teams at IHI. The frequency of future meetings and training sessions will be determined by any need identified by either the CAB or IHI and by future research projects.

## Discussion

Most community advisory boards in developed countries have been established by lobbyists and activists with a specific agenda [1-6]. While in developing countries CAB formation is often driven by the scientific community itself [7-10]. Because of this fundamental difference, establishment of a productive CAB within a low-income, low-resource environment presents many unforeseen obstacles and challenges. Financial support, how to ensure proper training of community members, and how to best foster independent thought and action are just a few of the issues that will arise during the set-up process [7,9,11,12]. Instilling a feeling of empowerment is the only way to ensure that the members will remain engaged and protect the interests of their communities.

The Bagamoyo District CAB was established by BRTC as a result of a perceived need for the public's involvement to ensure successful, sustainable research within the community. The Bagamoyo CAB was not created for just one specific project or one clinical trial. Our goal was to establish a non-specific CAB capable of working alongside BRTC on any research-related activities involving the community. While still young, the Bagamoyo CAB is stable and energized for its role as community liaison of BRTC's research initiatives. Here we discuss some important issues to be considered when establishing a CAB in a resource-poor, low-education setting.

Working in a poor community introduced the unique challenge of monetary expectations of CAB representatives. From the beginning, BRTC has paid the travelling expenses (fare and pocket money) for the CAB members to attend all CAB related events. As small as the payment may appear, it is actually quite substantial for some of the members in our area. We are acutely aware that this financial support could influence CAB members to alter their convictions or opinions of the research projects.

However, at the present time, the financial backing of the CAB is necessary. Unlike the early editions of the CAB in California [1-6] where the community demanded that they have a role in research and public health concerns, the Bagamoyo community has not yet progressed to that point. Few in the community perceive or understand the value of their involvement in the important public health issues such as malaria, HIV/AIDS, or childhood illnesses. Thus, to expect the community and/or the representatives to pay for participating on a Board that they have yet to fully embrace is not reasonable on our part. We hope that our efforts in supporting the CAB and our continuing commitment to both enabling and empowering its members by whatever means necessary will hasten their realization that community involvement is the quickest way to elicit positive change. The ultimate goal is that the Bagamoyo CAB will become a distinct entity completely independent of BRTC and able to engage the public health matters that are important in their community.

One of the biggest challenges we faced in the training stage was the communication of complex and difficult scientific concepts to an audience lacking any basic science knowledge. In our case, most of our members had only a primary school education. Hence, the need for well-planned and thought-out CAB training cannot be over emphasized. We took great care to ensure that the concepts were understandable, while at the same time making sure that the simplified ideas retained their scientific accuracy. Where this was not an issue in our case, we could foresee problems arising if the researchers are not fluent in local language. Dedication and patience are absolutely necessary to ensure proper communication of information across the often sizable educational and linguistic divide between scientist and community.

Another important lesson we learned was that the role of the CAB member should be made clear to all those involved as early as possible. We defined the role of the members to be mediators of concerns, disseminators of information, and facilitators of interactions – the human link between BRTC and the community. They were never to play any role in our recruitment activities, in the ethical review of our research (i.e. not an additional review board) or in the design of any project or trial. We believe our initial failure to do so may have led to the large numbers of village political leaders in the first edition of the CAB, as they thought they would have a direct input to how our research was performed.

## Differences between the CAB and IRB in consultation of the community

The community consultation function of the CAB differs from IHI's IRB in two major ways. First, the CAB has an purely an advisory role, while the IRB has a legal mandate to approve or withhold approval of research on either scientific or ethical grounds, something the CAB has no mandate to do. The CAB can only suggest changes to IHI-BRTC research protocol. Second, issues raised by the CAB have been and, will likely continue to be, specific to local social and cultural ramifications, whereas the IRB must take into account both national and international norms in all discussions. While the CAB and the IRB differ in their perspective and legal power to affect change in research protocol, both help in their own way to protect the individuals and communities taking part in IHI-initiated research projects.

## Conclusion

Successful establishment of a community advisory board is a complicated process. It cannot be easily distilled in a "To Do" list or a specific list of rules. The challenges and obstacles will vary depending on the community, the research institute, and the reasons behind wanting to establish a CAB. From our experience, flexibility, patience, an ability to stop and reassess problems, and an unwavering commitment were the primary ingredients in the success we achieved with the Bagamoyo CAB thus far.

Yet, we also recognize that our efforts are far from being accomplished. We must continue to work to ensure that our past achievements translate to a future where the CAB is an independent, community-led organization. We hope that the Bagamoyo CAB will soon serve as a platform from which the community can take ownership of the many health issues that affect their lives daily.

## Competing interests

The authors declare that they have no competing interests.

## Authors' contributions

SA, KS, OJ, and RS were involved in establishing the CAB. SA, KS, and BB assessed the results and findings. BB drafted the manuscript. All authors reviewed and approved the final draft.
